# MiRNAs: a call to arms that shapes the plasticity of tumor associated macrophages in breast cancer

**DOI:** 10.3389/fimmu.2026.1792965

**Published:** 2026-02-18

**Authors:** Valentina Fogazzi, Giulia Cosentino, Michele Sommariva, Angela Galardi, Elisa Dell’Orto, Serenella M. Pupa, Cristian Taccioli, Marilena V. Iorio

**Affiliations:** 1 Microenvironment and Biomarkers of Solid Tumors Unit, Department of Experimental Oncology, Fondazione IRCCS Istituto Nazionale dei Tumori di Milano, Milan, Italy; 2 Department of Biomedical Sciences for Health, University of the Study of Milan, Milan, Italy; 3 Department of Animal Medicine, Production and Health, University of Padova, Padua, Italy; 4 Department of Computer Science and Technology, University of Cambridge, Cambridge, United Kingdom

**Keywords:** breast cancer, immune evasion, microRNAs, tumor microenvironment, tumor-associated macrophages

## Abstract

Breast cancer (BC) remains a leading cause of cancer-related mortality, and a major contribution to tumor progression and resistance to therapies arise from tumor microenvironment (TME). Tumor is indeed able to shape a self-permissive TME, reprogramming the cellular components into allies. Tumor-associated macrophages (TAMs), abundant in BC TME, mainly acquire an immunosuppressive M2-like phenotype able to fuel tumor progression, immune evasion, metastasis and therapy resistance through a dynamic crosstalk with cancer cells. MicroRNAs, transferred via extracellular vesicles and exploited by the tumor to mold an immunesuppressive niche, act as central mediators of this bidirectional communication: tumor-derived miRNAs can reprogram macrophages toward an M2-like functional program, and TAM-derived miRNAs in turn promote and sustain cancer cell progression. This miRNA-orchestrated plasticity highlights TAMs as key TME regulators. Clinically, miRNA modulation offers promising strategies for TAM reprogramming, alongside their utility as prognostic biomarkers. Integrating miRNA-targeted TME interventions with conventional therapies holds the potential to overcome resistance in high-TAM BC subtypes.

## 1 Introduction

Despite major breakthroughs in therapeutic strategies, breast cancer (BC) remains the leading cause of cancer-related death among women worldwide (1, 2). BC is a highly heterogeneous disease classified into distinct intrinsic molecular subtypes based on hormone receptors (ER and PR), and Human Epidermal Growth Factor Receptor 2 (HER2) expression: Luminal A, Luminal B, HER2-enriched, and Basal-like mainly represented by triple-negative breast cancer (TNBC) (3–5). Personalized treatment approaches have dramatically improved the patient outcome (6), however long-term disease control remains a major issue due to intrinsic or acquired therapeutic resistance and/or the lack of effective targeted therapies as in the aggressive TNBC (7). These limitations highlight the urgent need of more effective and complementary therapeutic strategies by exploring novel vulnerabilities. In this context, a notable example is the tumor-supporting behavior of tumor microenvironment (TME), that has moved beyond a cancer cell–centric view toward a more holistic perspective (8). Indeed, the tumor represents a complex ecosystem where cancerous cells are surrounded by an heterogeneous network of stromal and immune cells, embedded within a dynamic extracellular matrix (ECM), comprehensively known as TME (9). Through a continuous bidirectional dialogue, TME components are recruited and corrupted by cancer cells to sustain tumor evolution, progression and therapeutic resistance, working as active regulators rather than passive bystanders. This crosstalk drives cellular plasticity: both compartments dynamically remodel their phenotype to keep adapting to environmental stress. Among the mediators of this interplay, microRNAs (miRNAs) have emerged as key regulators capable of modulating gene expression in both cancer and TME cells, orchestrating reciprocal pro-tumoral reprogramming (10). In BC, TME is crucial in disease initiation, progression and therapeutic response (11). Moreover, the crosstalk between cancer and TME cells shapes a tumor-permissive milieu through the polarization of immune cells toward an immuno-suppressive phenotype, thus promoting immune evasion. Among immune cell populations in the BC TME, tumor-associated macrophages (TAMs) are one of the most abundant, especially in TNBC, where higher TAM infiltration is associated with poorer prognosis (12–15). TAM involvement in BC progression underscores the crucial contribution of non-cancer cell components in shaping BC biology and highlights the therapeutic potential of targeting TME-associated circuits as miRNA-mediated crosstalk, a still not fully explored opportunity.

## 2 Macrophage plasticity and TME reprogramming in BC

Macrophages are innate immune cells fundamental for tissue homeostasis, host defense, and inflammatory response (16). They are widely distributed as tissue-resident populations in virtually all organs, while their circulating precursors, monocytes, patrol the bloodstream (17, 18). They can be recruited into tissues to reinforce local immune responses, where they differentiate into macrophages, acquiring adhesion properties and enhanced phagocytic capacity. Functional plasticity represents a defining hallmark of macrophages (19). Indeed, they can dynamically adapt their phenotype and functions to local microenvironmental signals. Cancer cells exploit macrophage plasticity inducing them to assume a pro-tumoral phenotype critical for tumor progression.

### 2.1 Macrophage classification

Macrophage activation has been historically categorized into two polarized states: M1 and M2 (20–22). Classically activated (M1) macrophages are induced by pro-inflammatory stimuli such as interferon-γ (IFN-γ), lipopolysaccharide (LPS) and other Toll-like receptor (TLR) ligands (21, 23, 24). They are characterized by elevated antigen-presenting capacity, potent microbicidal and tumoricidal activity, a strong pro-inflammatory profile through production of cytokines including TNF-α, interleukin-1 beta (IL-1β) and IL-6, reactive oxygen and nitrogen species, and typically express markers such as iNOS and MHC II. Functionally, M1 macrophages contribute to host defense, pathogen clearance, inflammatory responses and adaptive immune response by activating T helper 1 (Th1) cells. Alternatively activated (M2) macrophages arise in response to anti-inflammatory signals such as IL-4, IL-13, IL-10, glucocorticoids, and TGF-β (21, 23, 24). They support tissue remodeling, wound healing, angiogenesis, and inflammation resolution, express markers such as arginase 1 (Arg1), CD163, and CD206, and produce anti-inflammatory cytokines including IL-10. Functionally, M2 macrophages are involved in immunoregulation, suppression of inflammation and tissue repair. Also, they reinforce Th2-type immunity limiting Th1-mediated activity. Beyond the classical M1/M2 dichotomy, the M2 compartment comprises heterogeneous subpopulations (M2a, b, c and d) defined by distinct activating cues and functional programs, such as tissue repair, immune regulation and pro-angiogenic activity, indicative of macrophage functional heterogeneity (25, 26). Recently, single-cell RNA-seq analysis of human BC TAMs identified seven distinct TAM subtypes, ranging from pro-inflammatory/anti-tumor to pro-tumoral functional state (12). These findings corroborate that the classic M1/M2 dichotomy is an oversimplification, as TAM phenotypes form a dynamic continuum with overlapping activities affecting inflammation, immune modulation, angiogenesis and tumor progression. Interestingly, the presence of pro-inflammatory TAM subsets highlights the controversial role of inflammation in cancer, which can both constrain and fuel tumor progression depending on context. This further supports the idea that macrophage activation does not occur in discrete polarization states but rather encompasses a dynamic and context-dependent spectrum of phenotypes that can shift in response to microenvironmental stimuli (27). However, the term “M2 phenotype” is commonly used to describe TAM populations with immunosuppressive and pro-tumoral functions.

### 2.2 How to become a TAM: recruitment at the tumor site and M2 polarization

In cancer, circulating monocytes are recruited into TME by soluble factors or extracellular vesicle (EVs)-loaded molecules released from tumor cells (28). Key chemoattractants include chemokines such as CCL2, CCL5 and CXCL12, growth factors like CSF−1 and CSF−2, and VEGFA (29, 30). Tumor−derived EVs further contribute to this process by delivering proteins and RNAs that enhance monocyte chemotaxis and survival (31). Major drivers of M2 polarization are anti−inflammatory cytokines and growth factors, including IL−4, IL−10, TGF−β, and CSF−1, secreted by tumor, stromal and immune cells within the TME (32). In parallel, hypoxic conditions in poorly vascularized tumor regions stabilize HIF−1α and HIF−2α in infiltrating macrophages, driving metabolic adaptation (33). In addition, highly glycolytic cancer cells produce metabolites such as lactate, which act as environmental cues (34). Moreover, tumor−derived EVs play a critical role in macrophage reprogramming by transferring miRNAs, lipids, and immunomodulatory proteins that alter their transcriptional and epigenetic landscapes, dampen inflammatory pathways, and consolidate immunosuppressive functions (35, 36). Functionally, TAMs promote tumor growth and progression through different mechanisms. They secrete cytokines and growth factors, including VEGF, PDGF, and TGF−β that directly stimulate angiogenesis, cancer cell proliferation and survival, contributing to tumor expansion and poor clinical outcomes (37). In addition, TAMs facilitate metastasis and invasion by secreting matrix metalloproteinases (MMPs) able to remodel the ECM and promoting epithelial−mesenchymal transition (EMT), migration and the formation of pro-metastatic niches (38, 39). They also contribute to therapy resistance by activating survival pathways, impairing drug penetration through ECM remodeling, and supporting cancer stem cell niches (40). A central role of TAMs is the establishment of an immunosuppressive milieu that weakens effective anti−tumor immunity. TAMs release indeed anti−inflammatory cytokines such as IL−10, TGF−β, and IL−6, inhibit cytotoxic CD8^+^ T cell function, and recruit regulatory T cells, thereby suppressing adaptive immune responses (41). Importantly, TAMs also modulate immune checkpoint pathways: they can induce and express PD−L1 and other inhibitory ligands, further dampening cytotoxic T cell activity and contributing to immune evasion (42). Overall, these observations highlight the crucial role of the cross-talk between cancer cells and TME in orchestrating TAM reprogramming and function, thereby influencing the biological and clinical course of the disease. Among the key signals involved in this fundamental bidirectional dialogue, miRNAs have emerged as critical mediators.

## 3 MiRNAs: central regulators of BC-TME cross-talk and tumor progression

miRNAs are short (19–25 nucleotides), single-stranded non-coding RNAs regulating gene expression at the post-transcriptional level. MiRNA binding generally results in translational repression or mRNA degradation (43, 44). In cancer, miRNA dysregulation arises from multiple mechanisms, including genetic alterations, defects in the miRNA biogenesis machinery, and epigenetic modifications leading to widespread perturbation of gene regulatory networks (45–47). Consequently, miRNAs are deeply implicated in tumor initiation and progression and, depending on their molecular targets, can function either as oncogenes or as tumor suppressors. Beyond their cell-intrinsic roles, miRNAs have emerged as critical mediators of intercellular communication within the TME (48). Indeed, tumor and TME cells, including macrophages, actively exchange miRNAs as aberrant molecular messengers. This crosstalk is largely mediated by EVs, which protect encapsulated miRNAs and enable their stable transfer both locally within the tumor niche and systemically to distant sites (35, 49). Interestingly, even though the molecular mechanisms are still not fully understood, miRNA packaging into EVs is selective and not random (50, 51). Thus, miRNAs are central regulators of BC biology, acting not only as intracellular modulators of oncogenic pathways but also as key signaling molecules that integrate tumor-intrinsic programs with dynamic communication networks across the TME.

### 3.1 MiRNAs in BC cells

**Figure 1:**
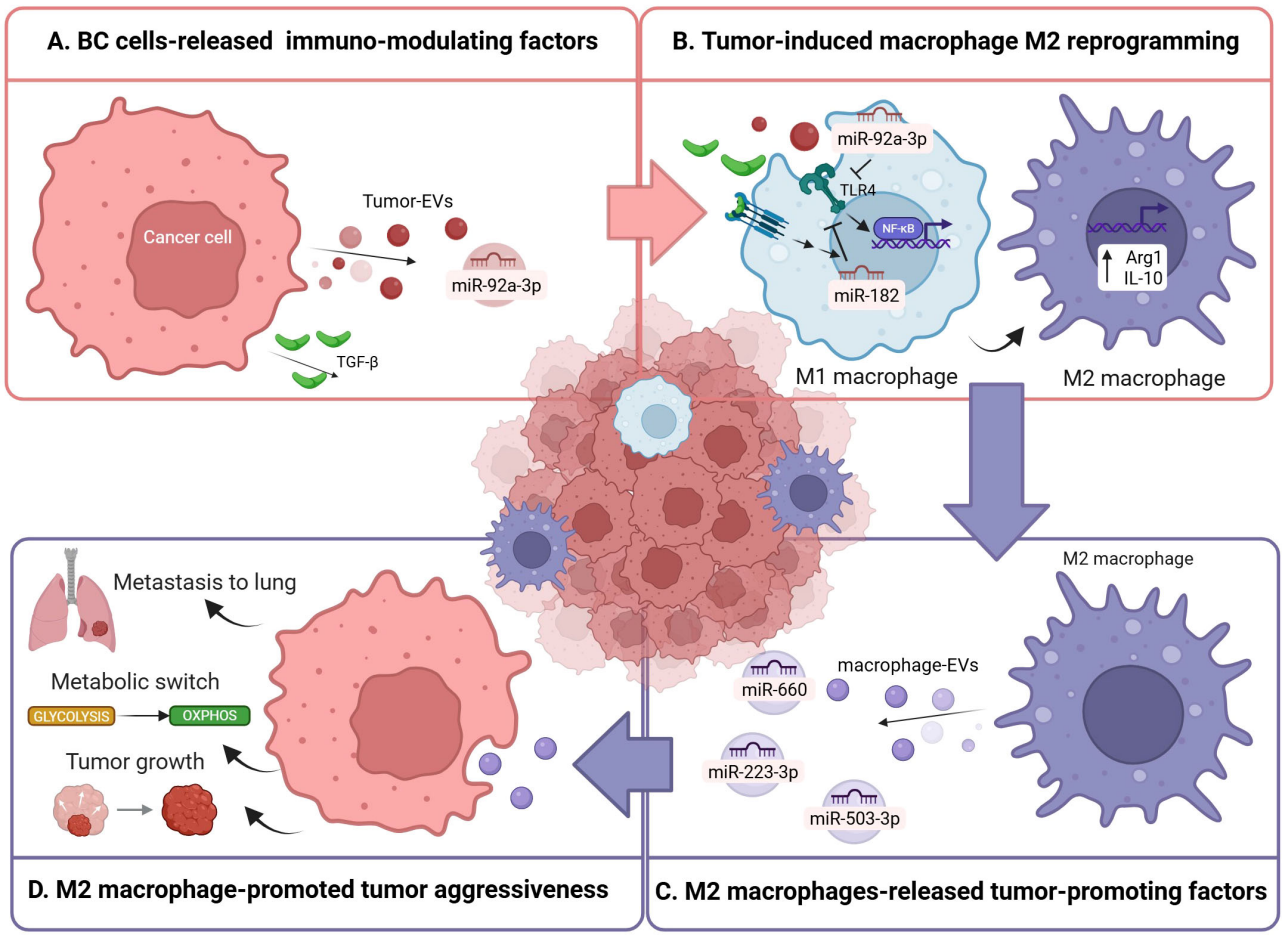
Graphical representation of the most recent findings on miRNA involvement in cancer cell-macrophage cross-talk in the BC microenvironment. In particular, miR-92a-3p and TGF-β are released by cancer cells **(A)** to induce an M2 macrophage reprogramming through the inhibition of TLR4/NF-kB signaling **(B)**. M2 macrophages, in turn, release miR-660, miR-223-3p and miR-503-3p **(C)** to induce, respectively, tumor growth, pro-tumoral metabolic switching and metastasis to lung **(D)**. Created with BioRender.com.

In BC, miRNA expression patterns correlate with key clinicopathological features such as hormone receptor status, lymph node involvement, proliferation index, and p53 status, highlighting their biological and clinical relevance (52). For instance, miR-29a promoted EMT, invasion, and lung metastasis by targeting PTEN and activating AKT signaling in ERα-positive luminal BC models both *in vitro* and *in vivo* (53). Response to therapy is also affected by miRNA dysregulation. Normann et al. detected improved response to the anti-HER2 treatment trastuzumab upon miR-101-5p overexpression and consequent alterations in key HER2 signaling-related pathways in HER2-positive BC cell lines, findings validated in TCGA and METABRIC datasets (54). MiRNAs can also play a tumor-suppressive role. Indeed miR-33b ectopic expression in HER2-positive BC resulted in a notable reduction in EMT, proliferation, invasion, and migration, while simultaneously promoting apoptosis; lower miR-33b levels in patient-derived tumor samples were associated with poorer survival (55). In addition, tumor cells exploit miRNAs as paracrine signals. Tumor-derived EVs carrying miRNAs contribute to mold a protumoral TME, including the reprogramming of macrophages toward an immunosuppressive M2 phenotype. EVs enriched with miR-92a-3p from BC *in vitro* models have been shown to be taken up by macrophages, where miR-92a-3p targets TLR4 thus increasing M2 markers and enhancing immune evasion and tumor invasiveness (**Figures 1A, B**) (56). Also, paclitaxel-resistant BC cells secrete exosomes enriched in miR-99b-3p, which enhances neighboring tumor cell migration and drug resistance by activating the AKT/mTOR pathway (53). Exosomal miR-99b-3p is also transferred to macrophages, where it promotes M2 polarization, indirectly reinforcing chemoresistance and invasiveness in drug-sensitive pre-clinical BC models both *in vitro* and *in vivo*. Moreover, tumor cells exhibit reduced release of tumor-suppressive miRNAs. For instance, hypoxic *in vitro* BC cells secrete exosomes with reduced miR-143-3p, leading to increased expression of a subunit of the mTORC2 complex in macrophages, which promotes an M2-like status and enhances BC cell invasion and migration (57). This is however a rare example because the current literature mainly describes miRNAs directly downregulated in TAMs and subsequently restored using engineered exosomes (58, 59). Moreover, tumor-suppressive miRNAs associated with favorable prognosis may indirectly influence immune–tumor cross-talk, but their specific roles in macrophages have not yet been fully elucidated (60). Overall, even though further investigation is certainly needed to elucidate how miRNAs influence the more complex and nuanced macrophage plasticity, all these studies illustrate a complex network in which BC cells actively modulate macrophage phenotype through miRNA-mediated signaling pathways, revealing exploitable targets for immunomodulatory therapies.

### 3.2 MiRNAs in TAMs

The modulation of miRNA expression within macrophages plays a central role in driving their phenotypic switch toward an M2-like state, by regulating signaling pathways and transcriptional programs (61, 62). For instance, miR-182 is induced in macrophages via tumor-secreted TGFβ signaling, where it downregulates TLR4 and promotes an M2-like functional program (**Figures 1A, B**); constitutive deletion of miR-182, as well as conditional knockout of miR-182 in macrophages, compromises M2-like TAM differentiation and limits BC progression in *in vivo* murine models (63). Conversely, miR-382 acts within macrophages to reduce M2 skewing by targeting metabolic regulators such as peroxisome proliferator-activated receptor gamma coactivator 1-alpha (PGC-1α), limiting the tumor-promoting functions of TAMs and consequently cancer progression and metastasis in *in vitro* and *in vivo* TNBC models (64). MiRNAs also serve as key messengers in TAM-tumor cell crosstalk (65). Indeed, TAMs actively promote BC progression through the secretion of EVs enriched with miRNAs that reprogram malignant cells. For instance, TAM-derived exosomal miR-660 promotes TNBC cell growth and metastasis through modulation of oncogenic signaling pathways such as the IκB kinase beta (IKKβ)/NF-κB p65 axis by direct inhibition of kelch like family member 21 (KLHL21), whereas miR-223-3p facilitates pulmonary metastasis by enhancing invasive and migratory capacities of TNBC cells in both *in vitro* and *in vivo* models (**Figures 1C, D**) (66–68). TAM signaling also contributes to metabolic plasticity, for instance macrophage-derived miR-503-3p suppresses glycolysis and promotes oxidative phosphorylation in breast cancer cells (**Figures 1C, D**) (69). Interestingly, Skourti E. et, by using dual radionuclide–fluorescence reporter system for spatiotemporal *in vivo* miRNA quantification, showed that miR-155 upregulation is dependent on macrophage infiltration, revealing immune-driven miRNA dynamics within the TME (70). Considering that miR-155 has also been reported as a LPS-induced pro-inflammatory miRNA in macrophages, it is clear that the effects mediated by miRNAs are context-dependent (71). Further highlighting the importance of miRNAs as key mediators of TAM–tumor cell crosstalk, exosomes from TAMs lacking protumoral progranulin are characterized by enhanced miR-5100 levels, a tumor-suppressor miRNA which directly suppresses the chemokine CXCL12 thus inhibiting BC cells invasion, migration, and EMT and reduce lung metastasis *in vivo* (72). Collectively, these studies underscore TAM-derived miRNAs as critical effectors of tumor-TME communication in BC, reinforcing an immunosuppressive and pro-metastatic microenvironment and identifying macrophage-derived miRNAs as potential therapeutic targets. However, the temporal dynamics and organization of these bidirectionality are still unclear and warrant further investigation.

## 4 Clinical perspectives of miRNAs in BC TAMs

Thanks to their intrinsic plasticity, migratory capacity, and phagocytic potential, macrophages have been harnessed to develop innovative cell-based cancer therapies. For instance, chimeric antigen receptor-macrophages (CAR−M) are engineered macrophages expressing synthetic receptors that enhance tumor recognition, phagocytosis, and pro-inflammatory activity (73). Preclinical studies have demonstrated that CAR−M can control tumor growth in solid tumors, including BC. This strategy exemplifies how macrophages can be exploited therapeutically, turning tumor-promoting populations into effector cells capable of orchestrating anti-tumor responses (74). The broad pro−tumoral activities of TAMs make them attractive unconventional therapeutic targets, and two main strategies have been explored in preclinical and clinical settings: depletion and reprogramming. Depletion approaches include targeting macrophage recruitment, survival, and growth signals, particularly targeting the CSF−1/CSF−1R axis (75, 76). In parallel, reprogramming strategies aim to shift TAMs from an immunosuppressive state to an anti-tumor phenotype using agents such as CD40 agonists, which activate macrophages and enhance their pro-inflammatory and tumoricidal functions, or inhibitors of immunosuppressive pathways (77). One of the promising avenues is TAM reprogramming through modulation of miRNA networks, since tumor and stromal signals can alter macrophage miRNA profiles. Conversely, restoring tumor-suppressive miRNAs or inhibiting pro-tumoral miRNAs in TAMs could shift the balance toward anti-tumor immunity (10). One of the major issues concerning miRNA-based therapy is considering the context-dependent biological effects and gene modulation, which requires a selective delivery to the cells of interest to limit side effects. For instance, Jing Z et al. tested a pH/ROS-responsive miR-155 nanocomplex designed to simultaneously reprogram tumor-infiltrating dendritic cells and TAMs in the TME to enhance anti-tumor immunity in TNBC (78). This dual reprogramming approach significantly increased CD8^+^ T cell infiltration, inhibited primary tumor growth, and reduced metastatic nodules *in vivo*, with minimal systemic toxicity. Ryu Y et al, instead, have recently proposed targeted restoration of miR-34a via anti-CD47 antibody–oligonucleotide conjugates which reactivates macrophage phagocytosis and boosts CD8^+^ T-cell responses, leading to tumor suppression in TNBC models (79). Similarly, ultrasound-triggered nanoparticles co-delivering paclitaxel and anti-miR-221 sensitize TNBC cells to chemotherapy while promoting TAM polarization toward an M1-like phenotype (80). The restoration of miR-299-3p impairs malignant behavior and simultaneously increases macrophage phagocytosis through dual targeting of CD47 and ABCE1, indicating a direct tumor-intrinsic and immune-modulatory mechanism (81). Beyond therapeutic modulation, miRNAs also have considerable value as biomarkers. Ray et al. developed integrated miRNA- and mRNA-based immune cell signatures in BC (82). This approach highlighted M2-like TAMs as strongly associated with poor prognosis, emphasizing that miRNA-mediated regulation of macrophages not only drives tumor-promoting phenotypes but can also serve as a prognostic biomarker within the TME. Moreover, a recent analysis of TCGA−BRCA cohort, validated in an independent clinical dataset (GSE22220), identified an 11−miRNA signature associated with TAMs, which stratifies BC patients into high− and low−risk groups for overall survival (83). These works highlight that specific miRNA profiles in TAMs not only reflect the immunosuppressive state of the TME but can also serve as clinically actionable biomarkers for patient stratification and survival prediction in BC, particularly in aggressive subtypes with high TAM infiltration. Certainly, as for any signature-based biomarker, standardization and normalization strategies need to be optimized.

## 5 Conclusion

TAMs are central orchestrators of BC progression, driving immune suppression, metastasis, and therapy resistance. In this review, we aim at focusing on miRNAs: despite the still open questions, miRNAs emerge s pivotal regulators of TAM plasticity, mediating both macrophage polarization and bidirectional communication with cancer cells, thereby influencing tumor progression at multiple levels. In addition, while therapeutic strategies targeting TAMs, through depletion, reprogramming, or CAR−M approaches, hold promise, miRNA modulation also represents a versatile and still not deeply explored avenue for intervention. Beyond their therapeutic potential, TAM-regulating miRNAs serve as actionable biomarkers for patient stratification and prognosis, offering a unique opportunity to integrate microenvironment-targeted approaches with conventional and immunotherapeutic strategies to achieve more precise and effective treatments, particularly in aggressive BC subtypes with high TAM infiltration.
